# Personal Portraits

**Published:** 1849-03

**Authors:** 


PERSONAL PORTRAITS.

In a complimentary notice of the letters of our Foreign Correspondent, the editors of the Medical Examiner make the following remarks respecting a practice which is but too common among travelers:

"Of the propriety and justice of describing personal appearance, peculiarities, and opinions, of living men, without their knowledge or consent, we have never been satisfied. Very often, the reporter has enjoyed but limited opportunities for judging of what he relates, and is, therefore, liable to commit gross, although it may be, unintentional errors; nor can any thing, in our opinion, justify the breach of confidence which is committed by those who disregard the sanctity of the fireside, and expose scenes which their eyes have been permitted to behold only from the promptings of a generous hospitality.

"In allusions of this kind, our young author has manifested a delicacy which contrasts favorably with the observations of some older tourists, who have preceded him in the same line of narration; generally, indeed, he has confined himself to a notice of professional rather than personal matters, and when speaking of the eminent men whom he saw, their opinions and practice on matters of professional interest alone are mentioned, and mostly, we think, in a way not calculated to offend."

The professional character of every man is public property. Our correspondent, as remarked by the editors of the Examiner, confined himself closely to professional topics, and whenever he alluded to personal peculiarities they were such as were brought out by the public position of the individuals.

